# A new species of Oriental-endemic *Thalerosphyrus* Eaton, 1881 (Ephemeroptera, Heptageniidae) from the Chinese Yunnan Oriental–Palaearctic transition zone and insights into cryptic diversity in the *T. flowersi* complex

**DOI:** 10.3897/zookeys.1272.181911

**Published:** 2026-03-02

**Authors:** Pandiarajan Srinivasan, Paul Tien Zhi Xian, Eleanor Tan Shu Ya, Yuchen Ang

**Affiliations:** 1 Department of Zoology, Sri S. Ramasamy Naidu Memorial College, Sattur-626203, India National University of Singapore Singapore Singapore; 2 Lee Kong Chian Natural History Museum, National University of Singapore, 2 Conservatory Drive, Singapore 117377, Singapore Department of Zoology, Sri S. Ramasamy Naidu Memorial College Sattur India

**Keywords:** COI, cryptic species, DNA-barcoding, Ecdyonurinae, Ephemeroptera, Heptageniidae, identification key, Oriental–Palaearctic transition, Oriental region, Southeast Asia, taxonomy, *Thalerosphyrus flowersi*, Yunnan

## Abstract

The Ephemeropteran genus *Thalerosphyrus* Eaton, 1881 (Heptageniidae: Ecdyonurinae) is an Oriental-endemic genus hitherto comprising ten species, distributed from Sundaland to the Western Ghats of India and northeastern Indochina. Here, *Thalerosphyrus lannaae***sp. nov**., belonging to the *T. sinuosus* group, is described from Yunnan Province (China), marking the northernmost record of the genus and extending its distribution into the Oriental–Palaearctic transitional zone. We also examined existing molecular data for *T. flowersi*, which revealed multiple deeply divergent lineages across India and Thailand, with the new species genetically closest to one of the Thai lineages. These findings highlight unrecognised cryptic diversity within the genus and underscore the need for taxonomic revision. An updated species key to *Thalerosphyrus* is provided. We discuss how larval preference for moderately cool, fast-flowing streams may explain the discovery of this tropically adapted Oriental-endemic genus in such high latitudes, and we explore the importance of transitional zones for aquatic insect diversity.

## Introduction

*Thalerosphyrus* Eaton, 1881 (Ephemeroptera: Heptageniidae: Ecdyonurinae) is a small Oriental-endemic mayfly genus currently comprising ten valid species: *Thalerophyrus determinatus* (Walker, 1853); *T. sinuosus* (Navás, 1933a); *T. vietnamensis* (Dang, 1967); *T. bishopi* Braasch & Soldán, 1986; *T. flowersi* Venkataraman & Sivaramakrishnan, 1987; *T. lamuriensis* Sartori, 2014a; *T. meghalayensis* Selvakumar & Chandra, 2017 (in Selvakumar et al. 2017); *T. thailandensis* Sutthacharoenthad, Sartori & Boonsoong, 2019; *T. bengalensis* Vasanth, Kubendran & Subramanian, 2025 and *T. sartorii* Vasanth, Kubendran & Subramanian, 2025.

Taxonomic understanding of *Thalerosphyrus* has undergone considerable revision due to challenges associated with its type species, *T. determinatus* (Walker, 1853): Initially described based on a single male imago from Java (Indonesia), the poorly preserved specimen lacked key diagnostic features that complicated its diagnosis (Kimmins 1960). These uncertainties led Kluge (2004) to treat the genus as *incertae sedis* until a comprehensive re-evaluation by Sartori (2014b), building on Ulmer’s (1939) earlier work, stabilised the genus. Larvae are readily recognisable by a distinctive suite of characters as described in Boonsoong and Braasch (2013): i) a thickened anterior margin of the head capsule; ii) long posterolateral spines on the abdomen; iii) sharply pointed supracoxal spurs; and iv) well-developed lamellae on tergalius I.

Hitherto, *Thalerosphyrus* has been recorded throughout Southeast Asia (up to the Sunda Islands) and India. Its presence on Sumbawa, east of the Wallace Line, demonstrates that this major biogeographic boundary does not restrict the genus’s distribution, paralleling patterns seen in several other heptageniid groups (Sartori 2014a). Historically, *Thalerosphyrus* was first described from the Tropical Sunda region, with *T. determinatus* and *T. sinuosus* recorded from Java and Sumatra, respectively. Subsequent discoveries expanded the genus’s distribution into Vietnam with *T. vietnamensis*, Peninsular Malaysia with *T. bishopi*, and southern India with *T. flowersi*. The northernmost records were established with *T. meghalayensis* from the East Khasi and East Jaintia Hills of Meghalaya, northern India. Most recently, two additional species (*T. bengalensis* and *T. sartorii*) were described from larvae collected in West Bengal and Tamil Nadu, respectively (Vasanth et al. 2025), further increasing the known diversity of the genus in the Indian subcontinent to four species. Recent studies have also suggested that some widespread species, particularly *T. flowersi*, may comprise multiple cryptic lineages (e.g., Vasanth et al. 2025), indicating that species boundaries within the genus are not yet fully resolved.

Hitherto, no nominal records of *Thalerosphyrus* have been documented in China. The species *Thalerosphyrus cingulatus* Navás, 1933b, originally described from China, was reassigned to the genus *Regulaneuria* Zhou, 2021 (in Lei et al. 2021). This reclassification was based on distinct morphological characters, including the fused penes of the male imago and the characteristic shape of the larval tergalii I–VII (Lei et al. 2021). In this article, we describe a new *Thalerosphyrus* species from Yunnan Province of China based on the larval specimens stored at the Lee Kong Chian Natural History Museum, representing the first valid and confirmed record of the genus in China and extending the known range of *Thalerosphyrus* further north into the transitional zone between the Oriental and Palaearctic realms.

## Material and methods

Specimens from a joint exploratory survey by the Lee Kong Chian Natural History Museum (**LKCNHM**; then known as the Raffles Museum of Biodiversity Research – RMBR) in 2000 (see Cheng et al. 2006) were analysed by the lead author SP, who identified two specimens belonging to the new species. Both specimens were collected using shallow-bottom sampling kick-nets (30 cm square with 2 mm mesh). Note that the collection details from Cheng et al. (2006) are updated here based on additional field notes from survey members Lanna Cheng (LC) and Tan Heok Hui (THH). Larval morphological characters of the new species were documented using two imaging systems: stereo images were acquired with a Leica M205C stereozoom microscope (Leica DMC5400 camera) and focus-stacked in Leica LASX core software. Slide-mounted specimens were acquired with an Olympus BX50 DIC compound microscope (Olympus DP275 camera) for slide images. Captured images were subsequently processed for publication using Adobe Photoshop 7.0. Non-destructive DNA barcoding was done as outlined in Yeo et al. (2021). Briefly, DNA extraction was conducted for individual specimens using HotSHOT buffers (Truett et al. 2000), amplified via PCR with tagged primers, cleaned, and sequenced with a MinION (Oxford Nanopore Technologies, Oxford, UK) to assemble 313 bp barcodes using ONTBarcoder (v.2.3.0; Srivathsan et al. 2023). Barcodes were then clustered via Objective Clustering (see Meier et al. 2006) to group specimens into MOTUs for morphological verification. Specimens are deposited in the Zoological Research Collection (**ZRC**) at the LKCNHM, Singapore.

## Results

### Thalerosphyrus lannaae

Taxon classificationAnimaliaEphemeropteraHeptageniidae

Srinivasan & Ang sp. nov.

4409812E-0653-594B-8ECB-7E4E29136DF6

https://zoobank.org/056D051A-84C0-4BA0-B841-2C5590936155

Figs 1, 2, 3, 4, 5, 6, 7, 8, 9, 10, 11, 12, 13, 14, 15, 16, 17, 18, 19, 20, 21, 22, 23, 24, 25, 26, 27, 28

#### Materials examined.

***Holotype*** (on slide): • 1 male larva (ZRCENT00020491); China, Yunnan, Mengla County [勐腊县], Mohan Town [磨憨镇], slightly muddy river by east of roadside, c. 2 km north of Shangyong Village [尚勇村]); 21°15'41"N, 101°42'58"E; ~735 m ASL; coll. Cheng L., 23 May 2000. GenBank Accession Code (PX640218). ***Paratype*** (in ethanol): • 1 female larva (ZRCENT00020492); 20 km north along highway G213 from SiMao District [思茅区], small clear flowing hill stream with sand and gravel bottom (pH 7.9), in ravine c. 450 m east of the highway; 22°53'66"N, 101°02'84"E; ~1400 m ASL; coll. Tan H. H., 20 May 2000.

**Figures 1–4.**
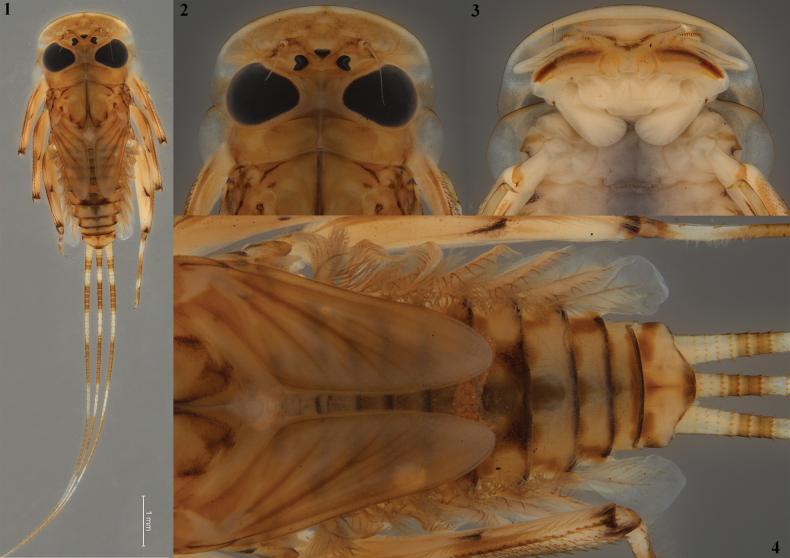
*Thalerosphyrus lannaae* sp. nov. male larva (holotype specimen ZRCENT00020491). **1**. Habitus, dorsal view; **2**. Closeup of head and thorax, dorsal view; **3**. Closeup of mouthparts and prosternum, ventral view; **4**. Closeup of tergum, dorsal view.

#### Diagnosis.

Larval diagnostic characters of *Thalerosphyrus lannaae* sp. nov. are as follows: i) hypopharyngeal superlinguae with long, simple setae extending to concave margin (Fig. 7); ii) maxilla with c. 16 comb-shaped setae on the crown (Fig. 11); iii) inner and outer margins of labial glossa straight near apex (Fig. 13); iv) pronotum slightly expanded laterally and posteriorly (Fig. 2); v) dorsal surface of hind femur with numerous distinctly pointed ‘arrow-shaped’ setae (Fig. 19); vi) abdominal posterolateral projections strongly developed, reaching maximum size on segment VIII (Fig. 23); and vii) tergalius I elongated and asymmetrical, c. 1.8× wider than long (Fig. 24).

**Figures 5–9.**
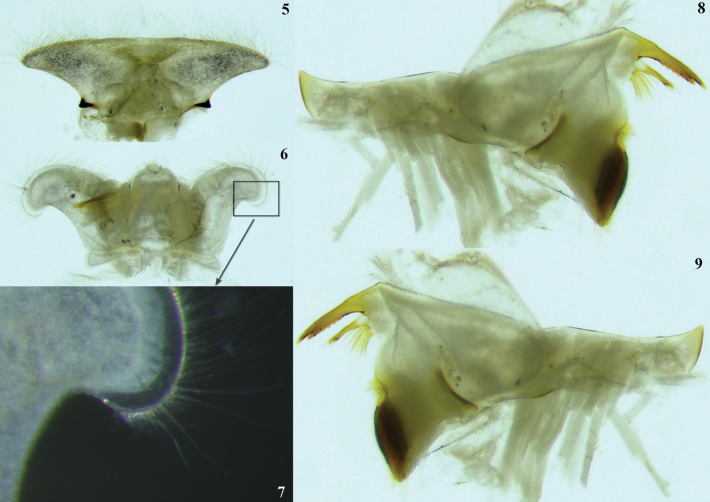
*Thalerosphyrus lannaae* sp. nov. male larva (holotype specimen ZRCENT00020491). **5**. Labrum, ventral view; **6**. Hypopharyx, dorsal view; **7**. Closer view of superlinguae, dorsal view; **8**. Left mandible, ventral view; **9**. Right mandible, ventral view.

**Figures 10–13.**
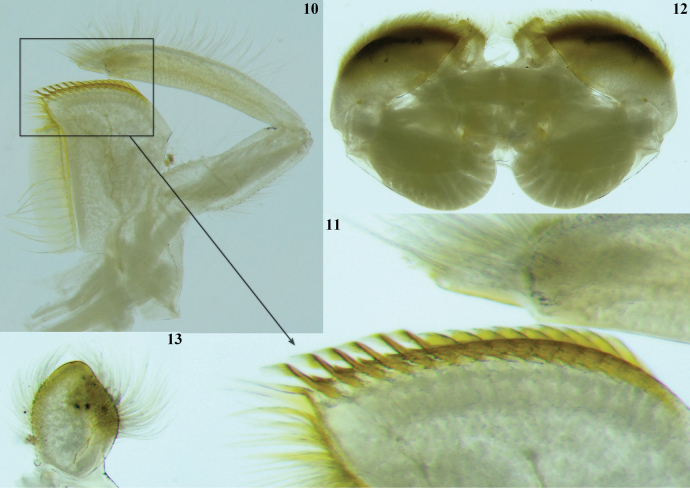
*Thalerosphyrus lannaae* sp. nov. male larva (holotype specimen ZRCENT00020491). **10**. Maxilla, ventral view; **11**. Closer view of the crown of maxilla; **12**. Labium, dorsal view; **13**. Glossa, dorsal view.

**Figures 14–17.**
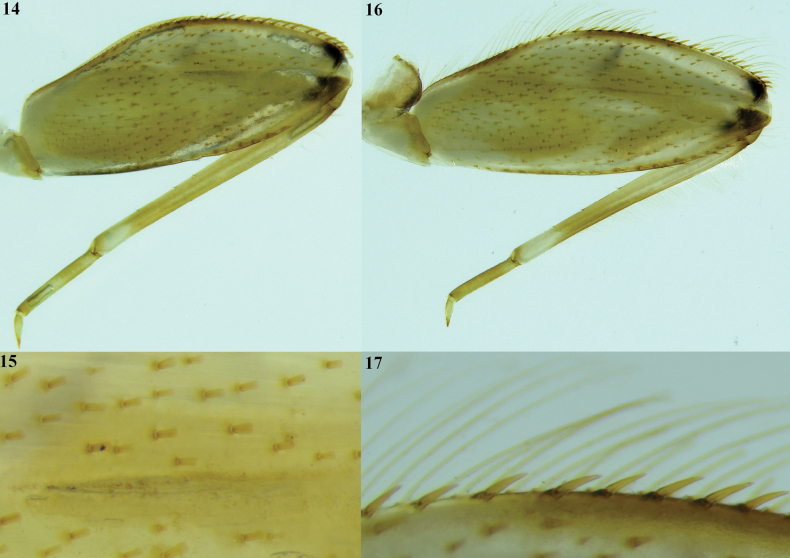
*Thalerosphyrus lannaae* sp. nov. male larva (holotype specimen ZRCENT00020491). **14**. Foreleg, dorsal view; **15**. Closer view of dorsal surface setae in forefemur; **16**. Midleg, dorsal view; **17**. Closer view of dorsal marginal setae in midfemur.

**Figures 18–22.**
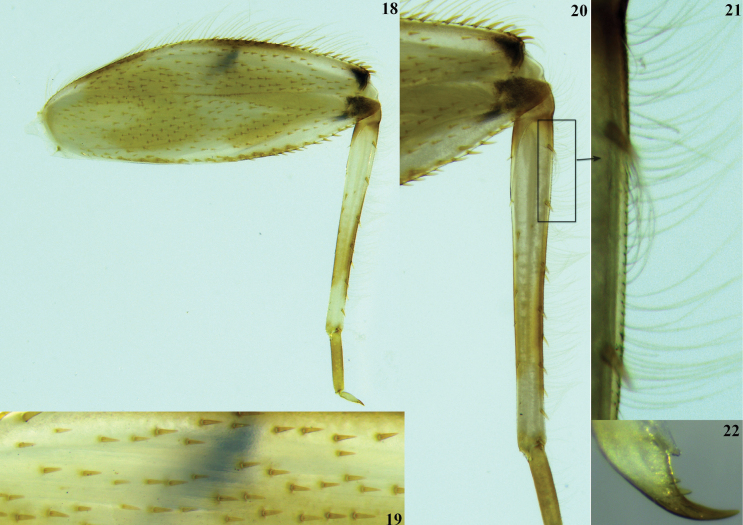
*Thalerosphyrus lannaae* sp. nov. male larva (holotype specimen ZRCENT00020491). **18**. Hind leg, dorsal view; **19**. Closer view of dorsal surface setae in hind femur; **20**. Hind tibia, dorsal view; **21**. Closer view of dorsal marginal setae in hind tibia; **22**. Hind claw.

**Figures 23–26.**
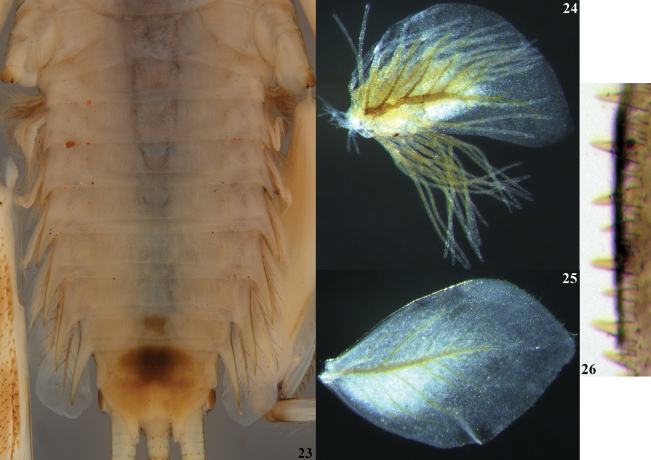
*Thalerosphyrus lannaae* sp. nov. male larva (holotype specimen ZRCENT00020491). **23**. Postero-lateral spines, ventral view; **24**. Tergalius I; **25**. Tergalius VII; **26**. Postero-tergal spines of segment IV.

#### Descriptions.

**Mature nymph. *Measurements***. Body length: 4.6–4.8 mm (Fig. 1), Cerci length: 6.5–6.7 mm.

***Coloration***. General coloration yellowish brown (Fig. 1). Head mostly yellowish, pronotum yellowish, anterior submedian region with dark brownish transverse band and mesonotum yellowish with sutures remain dark brownish (Fig. 2). Femur of all legs with dark brownish maculae near the distal apex, tibia of all legs pale and medial part remains yellow. Abdominal terga mostly yellowish brown, terga I–V dark brownish, tergum VI with conspicuous dark brownish posteromedial stripes, terga VII–X anterior margin with diffusive light brownish streaks at medial, submedial, and lateral areas (Fig. 4); sterna light (Fig. 3), with dark brownish markings medially in sternum IX. Caudalii coloration interchanges from pale to dark brownish band for every 4–5 segments (Fig. 1).

***Shape and setation*. Head. *Labrum***. Laterally elongated, c. 3.6 times wider than long, anterolateral margins elongated, tapering into a somewhat smooth rounded apices; dorsal surface and anterior margin with numerous long, thin hair-like setae (Fig. 5). ***Hypopharynx*** (Fig. 6). Lingua with a tuft of few small, simple setae medially; superlinguae with long, simple setae up to the concave margin (Fig. 7). ***Left mandible*** (Fig. 8). Inner margin of incisor serrated with c. 7 teeth; kinetodontium trifid or quadrifid, apically with few setae and below the inner margin c. 8 fimbriate setae present. ***Right mandible*** (Fig. 9). Inner margin of incisor serrated with c. 9 teeth; kinetodontium trifid, apically with few setae and below the inner margin c. 11 fimbriate setae present. ***Maxilla*** (Fig. 10). Galea-lacinia with long, thin simple setae across the inner margin; crown with c. 16 comb-shaped setae (Fig. 11); maxillary palp three segmented; maxillary palp segment I with a row of few small, thin, simple setae on the inner margin and outer margin with rows of long, simple setae; segment III subtriangular and bluntly pointed in the apex. ***Labium*** (Fig. 12). Glossae rhomboid; inner and outer margin remains straight near the apex (Fig. 13). ***Thorax***. Pronotum wider than the head, slightly expanded posteriorly and laterally, much wider than the head and basally fused to the mesonotum (Fig. 2). ***Legs***. Forefemur (Fig. 14) with a submarginal row of stout, spine-like setae on the distal 3/4^th^ area of outer margin and inner margin entirely covered with a submarginal row of stout, spine-like setae; dorsal surface with numerous spatulate setae (Fig. 15). Foretibia mostly bare in the outer margin and inner margin with 4–5 small, spine-like setae. ***Midfemur*** (Fig. 16) with submarginal rows of stout, spine-like setae on both outer and inner margins (Fig. 17). Midtibia with a row of hair-like setae on the outer margin and inner margin with 3–4 small, spine-like setae. ***Hind femur*** (Fig. 18) with submarginal row of stout, spine-like setae on both outer and inner margins, dorsal surface with clearly pointed arrow-shaped setae (Fig. 19). ***Hind tibia*** (Fig. 20) with a row of c. 3 stout, spine-like setae in the outer margin along with a row of thin setae in marginal position (Fig. 21) and submarginal area with c. 6 stout, spine-like setae. All tarsi mostly bare, with outer margins entirely covered with dense setation; tarsal claw with 3 small denticles (Fig. 22). ***Abdomen***. Posterolateral projections absent on abdominal segment I, weakly developed on segments II–IV, moderately developed on segment V and reaching their maximum size on segment VIII (Figs 23, 28). ***Tergalii***. Tergalius I elongated and asymmetrical, c. 1.8 times wider than long (Fig. 24); tergalii II–VI asymmetrical and wider than long; tergalius VII oval and symmetrical (Fig. 25). Posterior margin of tergite IV with irregularly pointed, small to long triangular spines and a few microdenticle rows (Fig. 26).

**Figures 27, 28.**
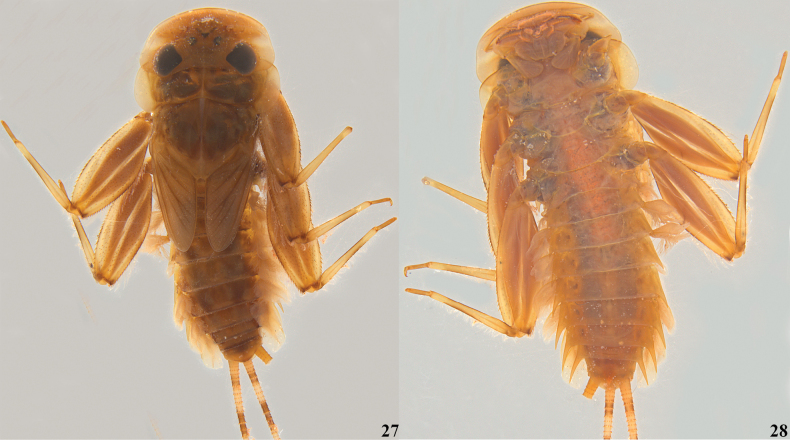
*Thalerosphyrus lannaae* sp. nov. female larvae (paratype specimen ZRCENT00020492). **27**. Habitus, dorsal view; **28**. Habitus, ventral view.

**Imago**. Unknown.

#### Etymology.

The new species is named after Dr Lanna Cheng, a marine insect specialist who was part of the Xishuangbanna (Yunnan, PR China) NUS Biological Expedition in 2000 and collected the holotype specimen of the new species.

#### Distribution.

China (Yunnan Province).

#### Taxonomic remarks.

*Thalerosphyrus lannaae* sp. nov. belongs to the *T. sinuosus* group (Braasch and Soldán 1984), characterised by elongated posterolateral projections on abdominal terga VI–VIII. Within the group, it shares two characters with *T. thailandensis*, *T. bengalensis*, and *T. sartorii*: (i) a reduced number of comb-shaped setae on the crown of maxillae and (ii) presence of only a few microdenticles on the posterior margin of tergite IV. The new species is distinguished from *T. thailandensis* by its strongly developed posterolateral projections on terga VI–VIII (moderately developed in *T. thailandensis*; Sutthacharoenthad et al. 2019: fig. 4c, to poorly developed in *T. meghalayensis*; Selvakumar et al. 2017: fig. 3), the presence of numerous pointed arrow-shaped setae on the dorsal surface of the hind femur (spatulate and rounded or truncate in *T. thailandensis*; Sutthacharoenthad et al. 2019: fig. 7d), and the long, simple setae on the superlinguae that extend to the concave margin (very small setae apically in *T. thailandensis*; Sutthacharoenthad et al. 2019: fig. 12e). Moreover, *T. lannaae* sp. nov. is distinguished from the other two aforementioned Indian species by the greatly developed posterolateral projections of the abdomen in *T. sartorii*, and by the distinctly concave margins of the glossae in *T. bengalensis*.

##### Key to the known larvae of *Thalerosphyrus* Eaton, 1881 from the Oriental region

**Table:** 

1	Hypopharynx with long setae reaching the concave margin of the superlinguae (Fig. 7; Sartori 2014a; fig. 28)	**2**
–	Setation of hypopharynx not as above (Sartori 2014a; Fig. 27; Sutthacharoenthad et al. 2019; fig. 2e)	**7**
2	Crown of maxilla with 16 comb-shaped setae (Fig. 11)	**3**
–	Crown of maxilla with more than 16 comb-shaped setae (Selvakumar et al. 2017; fig. 8)	**5**
3	Outer and inner margins of glossae concave (Vasanth et al. 2025; fig. 24)	***T. bengalensis***
–	Outer and inner margins of glossae nearly straight (Fig. 13)	**4**
4	Posterolateral projections of the abdomen greatly developed (Fig. 23)	***T. lannaae* sp. nov**.
–	Posterolateral projections of the abdomen moderately developed (Vasanth et al. 2025; fig. 54)	***T. sartorii***
5	Outer margin of hind tibia with a row of hair-like setae (Sutthacharoenthad et al. 2019)	***T. vietnamensis***
–	Outer margin of hind tibia with a row of pointed setae (Sartori 2014a; fig. 34)	**6**
6	Tergalius I asymmetrical and ovoid (Vasanth et al. 2025; fig. 107)	***T. flowersi***
–	Tergalius I elongated and forms rounded plate (Sartori 2014a; fig. 46)	***T. lamuriensis***
7	Tergalius I ratio: length more than 2.5 times its width (Sartori 2014a; fig. 38)	***T. determinatus***
–	Tergalius I ratio: length less than 2.5 times its width (Sartori 2014a; fig. 42)	**8**
8	Dorsal surface of hind femur with clearly pointed arrow-shaped setae (Sartori 2014a; fig. 31)	***T. sinuosus***
–	Setation of dorsal surface of hind femur not as above (Sartori 2014a; fig. 33; Sutthacharoenthad et al. 2019; fig. 7a, d)	**9**
9	Outer and inner margins of glossae nearly straight (Sutthacharoenthad et al. 2019; fig. 6d)	***T. thailandensis***
–	Outer and inner margins of glossae concave (Selvakumar et al. 2017; fig. 10)	***T. meghalayensis***

## Discussion

### Reassessment of *T. flowersi* sensu lato using molecular evidence

Examination of existing molecular evidence indicates that *T. flowersi* represents a species complex distributed across India and Thailand; a pattern independently corroborated by Vasanth et al. (2025), who noted clear morphological discrepancies between Thai material and true *T. flowersi*. In Suttacharoenthad et al. (2019), Thai specimens from Chiang Mai and Nan were identified as *T. flowersi*, with two individuals from each locality sequenced, one larva imaged, and an extended diagnosis provided. However, that diagnosis is difficult to interpret because it is unclear i) which specimens contributed to the morphological description, ii) which locality the imaged larva originated from, and iii) whether the Chiang Mai and Nan specimens were morphologically distinguishable. As such, their diagnosis may represent only one lineage, or a composite of both. The omission of published barcodes for Indian specimens from their analysis further restricted their ability to assess species boundaries. Their phylogenetic analysis, conducted in MEGA using NNI-based maximum-likelihood searches, recovered the Chiang Mai and Nan specimens as a single lineage sister to *T. thailandensis*, but the substantial genetic divergence between the two Thai localities was not examined further.

**Figure 29.**
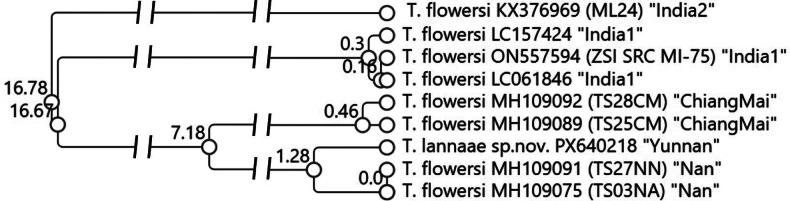
Dendrogram of *T. flowersi* and *T. lannaae* sp. nov. based on COI barcodes extracted from GenBank and the new species barcode from this study. Labels indicate species name, GenBank accession number, and available specimen codes (in brackets), followed by the designated MOTU in quotation marks. Node values represent uncorrected p-distances (%) where approximately 0.3% ≈ 1 bp.

Here, we compiled all publicly available *T. flowersi* COI sequences, incorporated COI data for *T. lannaae* sp. nov. and applied objective clustering (Meier et al. 2006). The resulting dendrogram (Fig. 29) reveals four clearly separated molecular operational taxonomic units (MOTUs): “India1”, “India2”, “ChiangMai”, and “Nan”, each differing by ≥ 7.18% uncorrected p-distance (≥22 bp), well beyond commonly applied species-level thresholds for insects. The two Indian MOTUs differ by 16.17%, indicating that only one corresponds to true *T. flowersi*, while the other represents an undescribed species. Likewise, the Thai specimens form two deeply divergent MOTUs, demonstrating that the Chiang Mai and Nan lineages constitute distinct species rather than a single taxon. *Thalerosphyrus lannaae* sp. nov. is genetically closest to the Nan cluster but differs by 1.28%, a separation just beyond the putative barcoding gap and, together with its diagnostic morphological traits, supports its recognition as a distinct species from the Thai specimens. Collectively, these results reveal substantial unrecognised diversity within *T. flowersi* sensu lato, with at least three undescribed *Thalerophyrus* species, and highlight the need for a comprehensive integrative revision of the species complex.

### Habitat associations and biogeographic patterns for *Thalerophyrus*

Studies of *Thalerosphyrus* habitat preferences indicate that stream quality, particularly oxygenation and substrate stability, is more critical than altitude. Research by Boonsoong and Braasch (2013) on the genus in Thailand demonstrated that *Thalerosphyrus* larvae are predominantly found in pristine montane and submontane rivers with high oxygen levels and stable substrates, such as gravel or sand. However, findings from Sundaic species (Hamid et al. 2016) and our present findings from Yunnan suggest that the genus is adaptable to lowland and mid-altitude streams, provided water quality remains high. Thus, while montane habitats offer optimal conditions, the genus’s ecological flexibility may explain its broader distribution.

The current distribution of *Thalerosphyrus* reflects the region’s geological and climatic history. Pleistocene climatic oscillations (~2.58 Ma–11.7 ka) played a significant role, with glacial maxima facilitating connectivity between the Sunda Shelf and mainland Southeast Asia via exposed land bridges (Voris 2000). Interglacial periods likely isolated populations in montane refugia, driving speciation. The Mekong River system, originating in the Tibetan Plateau and flowing through Yunnan, has likely acted as a dispersal corridor, enabling the northward expansion of *Thalerosphyrus* into transitional zones.

The discovery of *T. lannaae* sp. nov. in Yunnan represents the northernmost Oriental record of the genus, extending its known range into the transitional zone between the Oriental and Palaearctic realms. The specimens originate from the Xishuangbanna region, a biogeographically important area at the interface of southern Yunnan and Indochina, reinforcing the significance of this transitional zone in shaping aquatic insect distributions. This finding refines our understanding of the genus’s biogeographic limits and highlights the value of studying museum material to elucidate the evolutionary and ecological drivers of diversity within *Thalerosphyrus* and related taxa.

## Supplementary Material

XML Treatment for Thalerosphyrus lannaae

## Additional information

### Use of AI

No use of AI was reported.

### Author ORCIDs

Pandiarajan Srinivasan https://orcid.org/0000-0001-8118-3256

Paul Tien Zhi Xian https://orcid.org/0009-0008-1199-756X

Eleanor Tan Shu Ya https://orcid.org/0009-0007-5760-3231

Yuchen Ang https://orcid.org/0000-0001-5889-018X

### Data availability

All of the data that support the findings of this study are available in the main text.
